# Repeated Measurement of the Intermountain Risk Score Enhances Prognostication for Mortality

**DOI:** 10.1371/journal.pone.0069160

**Published:** 2013-07-17

**Authors:** Benjamin D. Horne, Donald L. Lappé, Joseph B. Muhlestein, Heidi T. May, Brianna S. Ronnow, Kimberly D. Brunisholz, Abdallah G. Kfoury, T. Jared Bunch, Rami Alharethi, Deborah Budge, Brian K. Whisenant, Tami L. Bair, Kurt R. Jensen, Jeffrey L. Anderson

**Affiliations:** 1 Intermountain Heart Institute, Intermountain Medical Center, Salt Lake City, Utah, United States of America; 2 Genetic Epidemiology Division, University of Utah, Salt Lake City, Utah, United States of America; 3 Cardiology Division, University of Utah, Salt Lake City, Utah, United States of America; 4 Department of Nutrition, Utah State University, Logan, Utah, United States of America; University Heart Center Freiburg, Germany

## Abstract

**Background:**

The Intermountain Risk Score (IMRS), composed of the complete blood count (CBC) and basic metabolic profile (BMP), predicts mortality and morbidity in medical and general populations. Whether longitudinal repeated measurement of IMRS is useful for prognostication is an important question for its clinical applicability.

**Methods:**

Females (N = 5,698) and males (N = 5,437) with CBC and BMP panels measured 6 months to 2.0 years apart (mean 1.0 year) had baseline and follow-up IMRS computed. Survival analysis during 4.0±2.5 years (maximum 10 years) evaluated mortality (females: n = 1,255 deaths; males: n = 1,164 deaths) and incident major events (myocardial infarction, heart failure [HF], and stroke).

**Results:**

Both baseline and follow-up IMRS (categorized as high-risk vs. low-risk) were independently associated with mortality (all p<0.001) in bivariable models. For females, follow-up IMRS had hazard ratio (HR) = 5.23 (95% confidence interval [CI] = 4.11, 6.64) and baseline IMRS had HR = 3.66 (CI = 2.94, 4.55). Among males, follow-up IMRS had HR = 4.28 (CI = 3.51, 5.22) and baseline IMRS had HR = 2.32 (CI = 1.91, 2.82). IMRS components such as RDW, measured at both time points, also predicted mortality. Baseline and follow-up IMRS strongly predicted incident HF in both genders.

**Conclusions:**

Repeated measurement of IMRS at baseline and at about one year of follow-up were independently prognostic for mortality and incident HF among initially hospitalized patients. RDW and other CBC and BMP values were also predictive of outcomes. Further research should evaluate the utility of IMRS as a tool for clinical risk adjustment.

## Introduction

The Intermountain Risk Score (IMRS) is a risk prediction tool created in a general medical population and validated in outpatient, inpatient, cardiovascular, and general populations [1]. IMRS has an exceptional ability to predict mortality and has broadened the understanding of the risk information in the complete blood count (CBC) and basic metabolic profile (BMP) [1]–[2]. For example, the study of the red cell distribution width (RDW) as a risk predictor arose from the development of IMRS (RDW is a component of IMRS) [3]. IMRS is an idealized clinical prediction rule because its components are well-established in medicine, are familiar to clinicians, are commonly ordered clinically, are quantitative assessments of the parameters they measure, can be entered into a risk score calculation outside of the clinic or hospital room (i.e., IMRS can be computed by laboratory equipment and from the hospital electronic medical record), and are relatively inexpensive tests that can be run at almost every medical center in the world [4]–[6].

IMRS utilizes all risk information from the CBC and the BMP to predict mortality, [1] and also predicts morbidities such as myocardial infarction (MI), heart failure (HF), stroke, and chronic obstructive pulmonary disease [2]. Its predictive ability for incident HF and HF-related outcomes is particularly strong [2]. Further, IMRS stratifies mortality risk not only overall but within each individual decade of adulthood and significantly predicts differences in life expectancies in each decade [7].

We hypothesized that longitudinal changes in patients’ IMRS are predictive of differences in mortality and cardiovascular risk. This study evaluated whether IMRS values measured after six to twenty-four months after an initial hospitalization are prognostic for mortality in the context of baseline IMRS.

## Materials and Methods

All adult (age≥18 years) female and male patients seen at Intermountain Healthcare hospitals between January, 1999, and January, 2009, were studied if they had both CBC and BMP laboratory panels performed at both a baseline hospitalization and a follow-up time point within 6 months to 2.0 years following hospital discharge (females: N = 5,698, males: N = 5,437). A similarly broadly-inclusive patient population was originally utilized to derive IMRS [1].

### Ethics Statement

This study was approved by the Intermountain Healthcare Urban Central Region Institutional Review Board as a minimal-risk general data-only project in which waiver of consent was granted by the Intermountain Healthcare Privacy Board. Because of the limited use of protected health information and the implementation of appropriate data safeguards, the study was determined by the Privacy Board to pose minimal risk to study subjects while its conduct would be impossible without access to the protected health data.

### Laboratory Testing

Patients’ IMRS values at the two time points were calculated using the two sets of laboratory measurements, patient age, and patient sex [1]. Mathematically, IMRS is a sex-specific linear combination of weighted regression coefficients for hematocrit, RDW, mean corpuscular volume (MCV), platelet count, mean platelet volume (MPV), mean corpuscular hemoglobin concentration (MCHC), white blood cell count (WBC), sodium, potassium, bicarbonate, creatinine, glucose, calcium, and age (see Appendix S1 for risk coefficients). CBC testing was performed using the COULTER Gen·S Hematology Analyzer (Beckman Coulter Corp, Hialeah, FL). The BMP panel was tested on the VITROS 950 clinical laboratory system (Ortho Clinical Diagnostics, Raritan, NJ). Of note, red blood cell count, hemoglobin, mean corpuscular hemoglobin, blood urea nitrogen, and chloride were excluded from IMRS models because those elements were multi-collinear with other CBC or BMP components (thus, if included in IMRS they would have artificially inflated the risk scores because they provided duplicate risk information).

Patients’ second (or follow-up) set of laboratory tests had to be performed no earlier than 6 months following baseline and no later than 2.0 years after the baseline date (mean time from baseline to follow-up testing was 1.0 year). Survival was evaluated using the follow-up time point as the index date since all patients had to survive to have a second set of laboratory tests to qualify for the study. Survival outcomes after the second laboratory test were evaluated during a longitudinal follow-up period of a mean of 4.0±2.5 years (range: 0.5–10 years).

### Statistical Considerations

Baseline and follow-up IMRS values were computed using the 1-year IMRS, which was originally derived to predict the endpoint of 1-year mortality (see Appendix) [1]. Other IMRS models were previously created to predict 30-day death or 5-year death, but given that all patients were followed for more than 30 days and the majority were followed for less than 5 years, the 1-year IMRS was used. The computation of the baseline IMRS entered age as the date at baseline hospitalization minus the patient’s birth date and the second IMRS calculation used patient age at the follow-up time (calculated as the age at baseline plus the amount of time until the follow-up laboratory test date). Based on prior work, [1], [3], [7] both baseline and follow-up IMRS were categorized into strata of low-, moderate-, and high-risk IMRS for specific analyses, which categories were defined as IMRS <9, 9–14, ≥15 for females and IMRS <11, 11–16, ≥17 for males. IMRS change (ΔIMRS) was computed as the difference between follow-up and baseline IMRS.

Follow-up and baseline IMRS (in univariable and bivariable analyses) were evaluated using Cox regression in sex-specific models to determine their association with survival outcomes. All-cause mortality (females: n = 1,255 deaths; males: n = 1,164 deaths) was designated as the primary study endpoint. Mortality outcomes were determined from hospital records, Utah death certificates, and the Social Security death master file. Secondary endpoints included: incident MI (females: n = 133 events; males: n = 147 events), incident HF admission (females: n = 168 events; males: n = 196 events), and incident stroke (females: n = 176 events; males: n = 148 events). MI, HF, and stroke were evaluated only for patients with no history of those events and the events were defined based on ICD-9 codes recorded on the discharge summary of the patient’s follow-up hospital encounter. Regression models evaluated follow-up IMRS along with baseline IMRS to determine their independent predictive ability. Further regression modeling evaluated subsets of patients by testing ΔIMRS among only those with low-, moderate-, or high-risk baseline IMRS.

Secondary hypotheses were also evaluated by Cox regression for ΔIMRS with adjustment for baseline IMRS, as well as for RDW to determine sex-specific survival for baseline and follow-up measurements of RDW adjusted for age and each of the other CBC and BMP components. Secondary hypotheses also included evaluating differences in ΔIMRS based on treatments at the baseline time point, including medical procedures and surgeries and discharge medications prescribed at baseline. A p-value ≤0.05 was considered significant for the primary test of hypothesis, while secondary hypotheses and analyses of secondary endpoints were not multiple-comparisons corrected but instead considered to be hypothesis-generating analyses.

## Results

Females (n = 5,698) averaged 61.8±18.1 years of age, and males (n = 5,437) were aged 61.2±16.3 years (p = 0.08). The distribution of females and males by age decade (Table 1) was also not different (p-trend = 0.11). IMRS was significantly higher in the population after a year of follow-up (Table 2) compared to baseline IMRS for both females and males. Table 2 also demonstrates, though, that changes during the period between the two laboratory measurement times exhibited a regression toward the mean wherein those with lower scores tended to experience risk score increases and those with higher scores tended to experience a decline of similar magnitude in their risk scores.

**Table 1:** Distribution of females and males by age decade.

Age category	Females (n = 5,698)	Males (n = 5,437)
18–29 years	6.80%	5.30%
30–39 years	6.90%	5.30%
40–49 years	10.90%	11.70%
50–59 years	15.90%	19.40%
60–69 years	20.00%	23.60%
70–79 years	22.50%	22.70%
≥80 years	17.00%	11.9%*

* p-trend = 0.11 for males vs. females.

**Table 2:** Mean IMRS at baseline and follow-up, and the change (ΔIMRS) over that time.

IMRS Measure	Females	Males
Baseline IMRS	10.6±4.8	12.2±4.2
Follow-up IMRS	10.9±4.9*	12.4±4.3*
ΔIMRS	0.2±3.6	0.2±3.9
ΔIMRS (Baseline low-risk)	1.6±3.3 (32.0%)	2.0±3.5 (35.6%)
ΔIMRS (Baseline moderate-risk)	0.2±3.5 (45.8%)	−0.2±3.6 (48.2%)
ΔIMRS (Baseline high-risk)	−1.6±3.4† (22.2%)	−2.5±3.6† (16.1%)

Data in parentheses are the percentage of patients in the corresponding baseline category.

* p<0.001 vs. baseline IMRS of the same gender,

† p-trend<0.001 (across low-, moderate-, and high-risk categories within the same gender).

### Association of IMRS with Mortality

Follow-up IMRS and baseline IMRS both predicted mortality (Table 3), and these separate risk score variables were independently predictive in a bivariable Cox model. Each incremental +1 risk score point of follow-up IMRS added 13% to a female’s and 14% to male’s risk of mortality, while each additional +1 point in baseline IMRS was associated with a 10% higher risk for females and 7% higher for males (Table 3). Adjustment for the amount of time between the baseline and second IMRS measurements did not alter these results more than small variation in the third decimal place of the hazard ratios.

**Table 3:** Association of follow-up IMRS and baseline IMRS, modeled as continuous variables, with mortality in Cox regression.

	*Females*		*Males*	
Variable	Hazard Ratio (95% CI)	p-value	Hazard Ratio (95% CI)	p-value
***Univariable models***				
Follow-up IMRS	1.20 per +1 increment (1.18, 1.21)	<0.001	1.18 per +1 increment (1.17, 1.20)	<0.001
Baseline IMRS	1.19 per +1 increment (1.17, 1.21)	<0.001	1.15 per +1 increment (1.14, 1.17)	<0.001
***Bivariable model***				
Follow-up IMRS	1.13 per +1 increment (1.11, 1.15)	<0.001*	1.14 per +1 increment (1.12, 1.16)	<0.001*
Baseline IMRS	1.10 per +1 increment (1.08, 1.12)	<0.001†	1.07 per +1 increment (1.05, 1.09)	<0.001†

* Adjusted for baseline IMRS;

† Adjusted for follow-up IMRS. CI: confidence interval.

**Figure 1:**
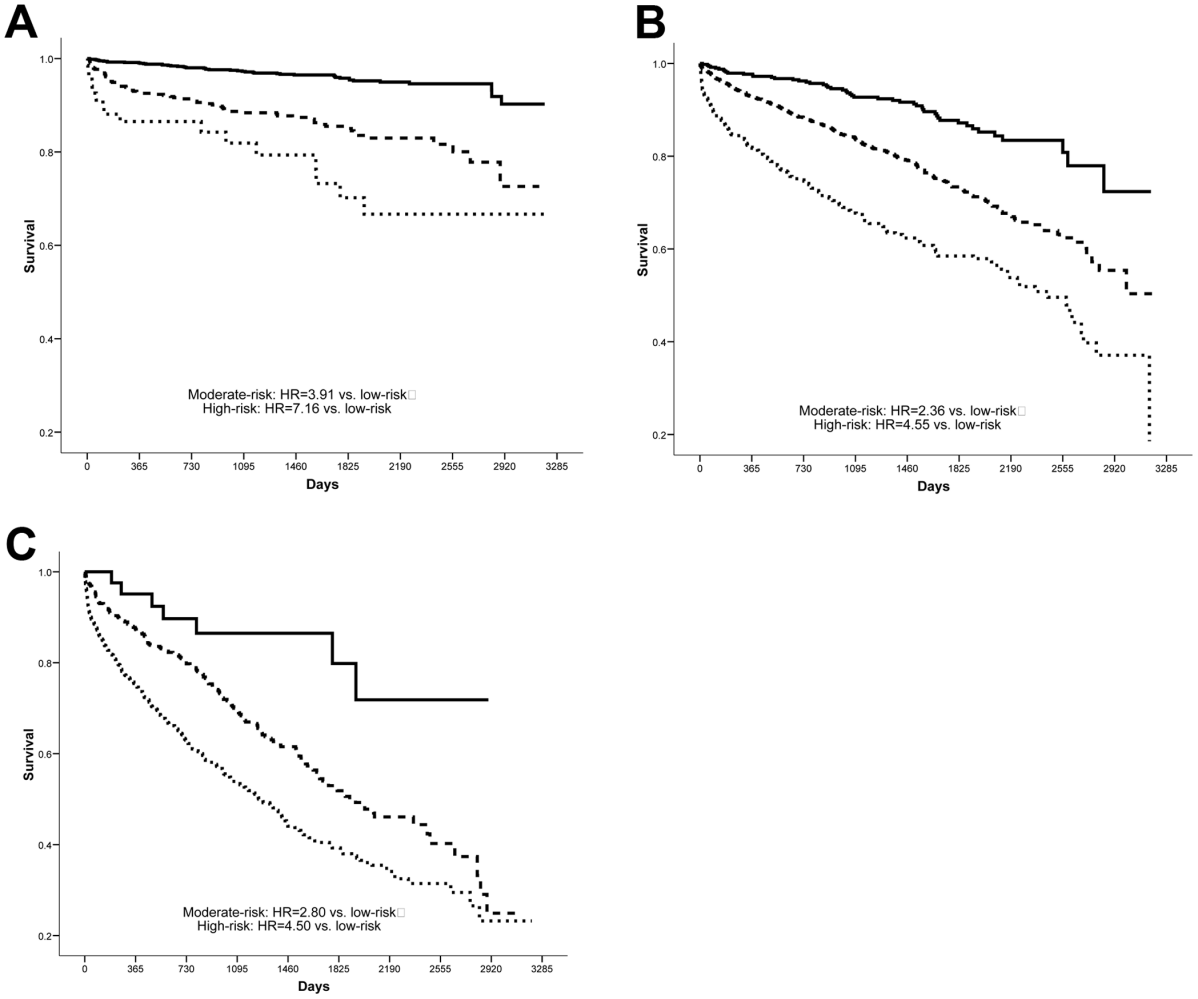
Females. Kaplan-Meier survival curves for the association of follow-up IMRS (low- [solid line], moderate- [dashed], and high-risk [dotted]) with mortality among females within strata defined by: **A**) low-risk baseline IMRS, **B**) moderate-risk baseline IMRS, and **C**) high-risk baseline IMRS (all p<0.001).

**Figure 2:**
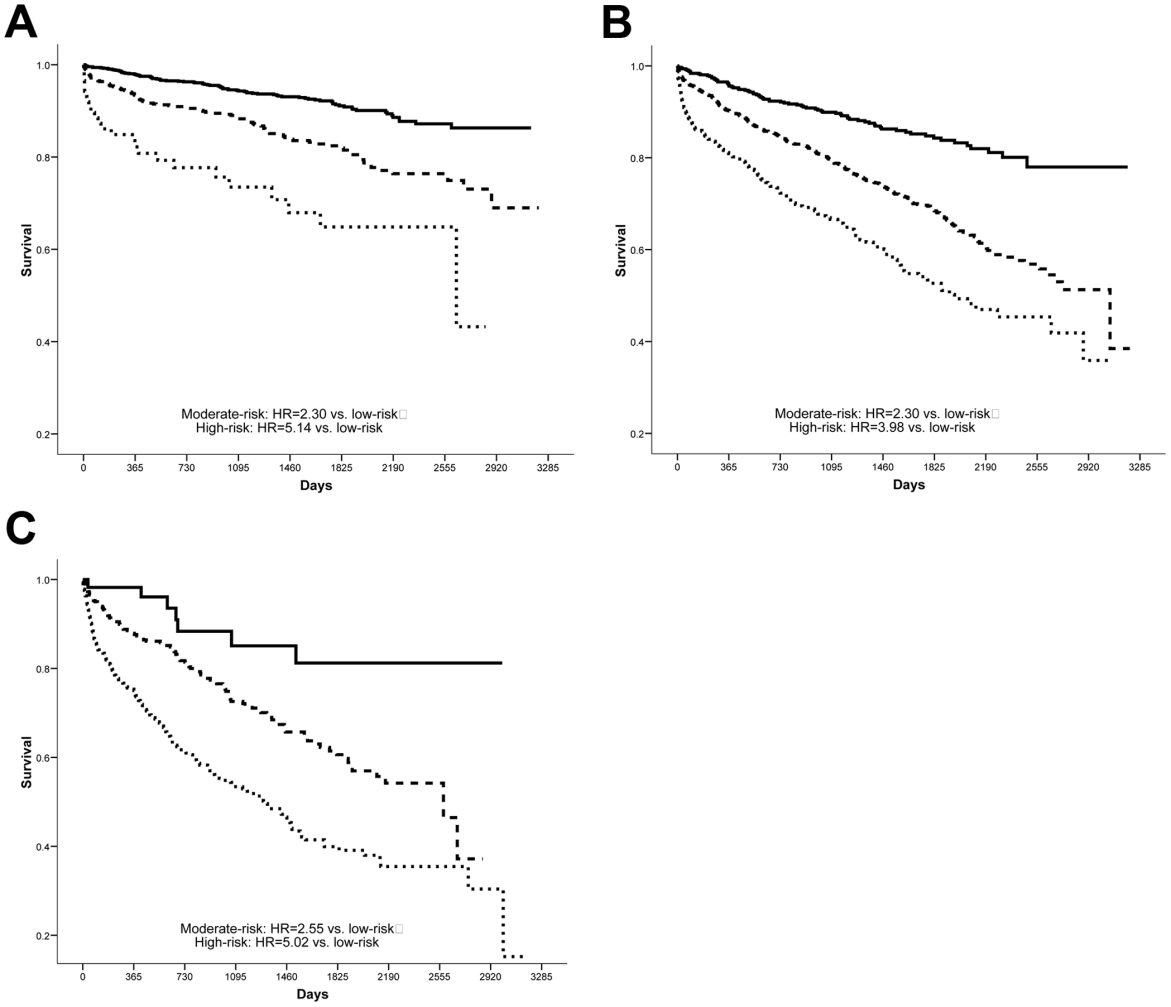
Males. Kaplan-Meier survival curves for the association of follow-up IMRS (low- [solid line], moderate- [dashed], and high-risk [dotted]) with mortality among males within strata defined by: **A**) low-risk baseline IMRS, **B**) moderate-risk baseline IMRS, and **C**) high-risk baseline IMRS (all p<0.001).

**Figure 3:**
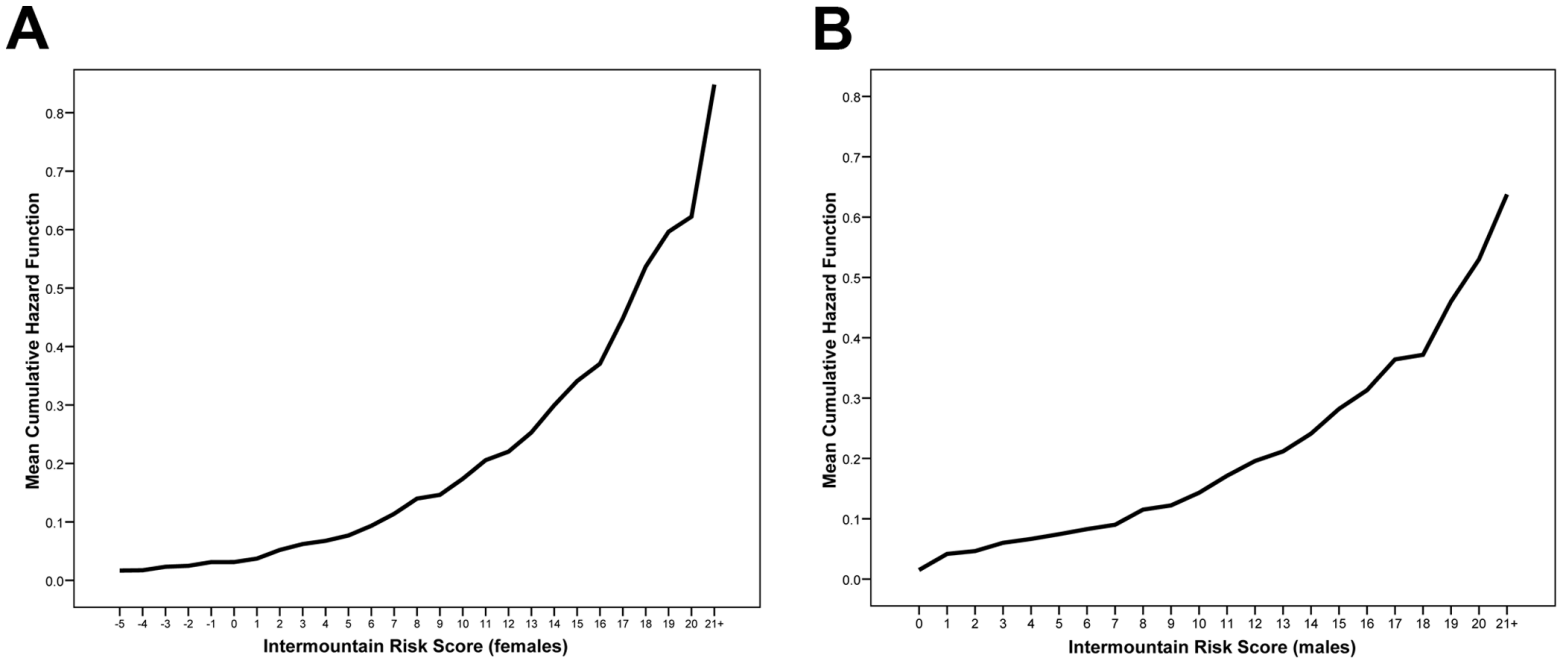
Average cumulative hazard function for mortality from Cox regression for A) females and B) males across each value of the second or follow-up IMRS.

When modeled as three categories of risk, both follow-up and baseline IMRS were independent predictors of mortality (all p<0.001 vs. low-risk). For females, a follow-up IMRS of moderate-risk had HR = 2.98 (CI = 2.37, 3.73) and the high-risk group had HR = 5.23 (CI = 4.11, 6.64) compared to low-risk, while baseline IMRS had significant but less pronounced risk prediction ability (moderate-risk group: HR = 2.07 [CI = 1.68, 2.54], high-risk: HR = 3.66 [CI = 2.94, 4.55]). Among males the results were similar, with a moderate-risk follow-up IMRS having HR = 2.32 (CI = 1.94, 2.77) and high-risk follow-up IMRS having HR = 4.28 (CI = 3.51, 5.22), while baseline IMRS had HR = 1.75 (CI = 1.49, 2.06) in the moderate-risk group and HR = 2.32 (CI = 1.91, 2.82) in the high-risk category. Figures 1 and 2 further highlight the independent contribution of both baseline and follow-up IMRS to the risk of mortality for females and males, respectively, by analyzing follow-up IMRS within strata defined by baseline IMRS. For follow-up IMRS, Figure 3 demonstrates the relationship of individual IMRS scores with the hazard of mortality.

Predictive ability for mortality of the baseline and follow-up IMRS were measured by ROC analysis and found to be greater for the follow-up IMRS (females: c = 0.761, males: c = 0.737) compared to baseline IMRS (females: c = 0.708, males: c = 0.666), although the baseline risk score still provided substantial risk information.

### Secondary Analyses

#### Delta-IMRS

IMRS decreased among 41.0% of females and 41.8% of males, while it was unchanged in 12.4% and 11.7% and increased among 46.6% and 46.5%, respectively. The change in IMRS from baseline to follow-up (ΔIMRS), adjusted for baseline IMRS, predicted mortality with an incremental 13–14% higher risk for each +1 additional IMRS point (females: HR = 1.11 per Δscore, 95% CI = 1.09, 1.13, p<0.001; males: HR = 1.12 per Δscore, 95% CI = 1.10, 1.14, p<0.001).

An increase in ΔIMRS was found in stratified analyses to contribute more to risk among those with an initially low IMRS than those with higher baseline risk, with HR = 1.20 per Δscore (CI = 1.15, 1.26) for females and HR = 1.14 per Δscore (CI = 1.10, 1.19) for males among patients with a baseline low-risk IMRS, compared to patients with a baseline moderate risk (females: HR = 1.12 per Δscore [CI = 1.09, 1.15]; males: HR = 1.12 per Δscore [CI = 1.10, 1.15]) and baseline high-risk IMRS (females: HR = 1.07 per Δscore [CI = 1.05, 1.10]; males: HR = 1.11 per Δscore [CI = 1.07, 1.15]). Dichotomous comparison of increased (ΔIMRS >3) to decreased IMRS (ΔIMRS<-3), excluding those with ΔIMRS of −3 to 3, showed substantially higher risk for females (HR = 1.35, CI = 1.11, 1.64; p<0.001) and even more so for males (HR = 1.64, CI = 1.34, 2.01; p<0.001).

Based on initial surgeries, procedures, and medications that were prescribed at baseline (Table S1), the results for ΔIMRS differed for some patient groupings (Table S2).

#### IMRS Associations with Major Causes of Death

Figure S1 shows hazard ratios for incident MI, HF, and stroke during longitudinal follow-up from models entering both baseline and follow-up IMRS. Among females (Figure S1A), a higher risk of MI was predicted by both baseline IMRS (moderate- vs. low-risk: p = 0.08; high- vs. low-risk: p = 0.021) and follow-up IMRS (p = 0.011, p = 0.07, respectively). Predictive ability of IMRS among females for stroke was also significant for both baseline IMRS (p = 0.07 for moderate-risk, p = 0.004 for high-risk) and follow-up IMRS (p = 0.001, p = 0.029, respectively). As in past analyses, [2] HF was the endpoint whose risk was best stratified by IMRS among females, again by both baseline IMRS (p = 0.001 for moderate-risk, p = 0.002 for high-risk) and follow-up IMRS (p<0.001, p<0.001, respectively).

Among males (Figure S1B), follow-up IMRS did not predict MI (moderate- vs. low-risk: p = 0.33; high- vs. low-risk: p = 0.60) and baseline IMRS was weakly predictive of MI (p = 0.044, p = 0.13, respectively). The results were similar for prediction of stroke (follow-up IMRS: p = 0.49 for moderate-risk, p = 0.70 for high-risk; baseline IMRS: p = 0.008, p = 0.042, respectively). For HF events, however, males were stratified well by IMRS (follow-up IMRS: p<0.001 for moderate-risk and p<0.001 for high-risk vs. low-risk; baseline IMRS: p = 0.024, p = 0.002, respectively).

#### RDW Associations

Multivariable Cox regression models entering both the follow-up and baseline CBC and BMP components for females (Table S3) and males (Table S4) showed that baseline RDW and follow-up RDW were independent predictors of mortality among both females and males. Various other components of the CBC and BMP panels were also independent predictors of mortality, although not necessarily at both time points.

Among females, the fifth quintile of baseline RDW (vs. quintile 1) predicted incident MI with HR = 2.06 (CI = 1.10, 3.85; p = 0.024), but follow-up RDW did not predict MI (p = 0.86), while quintile 5 vs. 1 of both follow-up RDW (HR = 2.28, CI = 1.05, 4.93; p = 0.037) and baseline RDW (HR = 2.30, CI = 1.08, 4.91; p = 0.031) predicted incident HF. Incident stroke was only predicted in females by follow-up RDW (quintile 5 vs. 1) with HR = 3.01 (CI = 1.46, 6.19; p = 0.003), and not by baseline RDW (p = 0.63).

For males, RDW did not predict MI at the follow-up (p = 0.86) or baseline (p = 0.52) time points, and follow-up RDW did not predict HF (p = 0.70), either, while baseline RDW (quintile 5 vs. 1) was predictive of incident HF (HR = 2.62, CI = 1.35, 5.10; p = 0.004). Incident stroke among males was also not predicted by follow-up RDW (p = 0.67) but was by baseline RDW (quintile 5 vs. 1: HR = 2.81, CI = 1.27, 6.23; p = 0.011).

## Discussion

IMRS utilizes common, familiar, and relatively inexpensive laboratory tests–the CBC and BMP–to summarize patient risk of mortality. Previous research showed that IMRS, measured at baseline, predicts the risk of all-cause mortality among general medical patients, higher-risk cardiovascular patients, and the general population, [1] and that IMRS predicts mortality during each decade of adulthood [7]. IMRS also predicts incident events including HF diagnosis, MI onset, and stroke among patients free from those events at baseline [2]. It also is associated with the presence of HF, MI, CAD, atrial fibrillation, chronic obstructive pulmonary disease, and peripheral vascular disease [2].

This study further revealed that repeated measurement of IMRS after approximately one year provides independent risk information beyond the baseline measurement of IMRS. Mortality risk for both females and males was highly significantly stratified by IMRS when measured at two time points among initially hospitalized patients. Both one-year follow-up IMRS and baseline IMRS were independently prognostic for mortality during a longitudinal period of up to 10 years after the second IMRS measurement. The change in IMRS was also associated with mortality independent of baseline IMRS. Further, baseline and follow-up IMRS measurements also predicted incident MI, HF, and stroke events in this study population. These findings indicate that IMRS is a powerful predictor of mortality and cardiovascular events when repeatedly measured.

The changes in IMRS itself may be of clinical significance in part because they may indicate changes in risk that have occurred during the follow-up period. In this study, patients with moderate- and high-risk IMRS at baseline who experienced a decrease in IMRS and who achieved a low-risk IMRS at follow-up had approximately the same level of mortality risk as those who had a low-risk baseline value and were in the low-risk level at follow-up. In contrast, patients who started in moderate- to high-risk IMRS categories at baseline and did not experience improvement in their IMRS, as well as those who progressed from low-risk to worse risk levels, had substantially worse outcomes than low-risk patients.

This risk information may be of substantial import for clinical risk stratification and chronic disease management because it demonstrates that risk that is revealed by IMRS is modifiable. This may encourage the re-measurement of IMRS on an annual basis, a clinically-relevant decision point that potentially provides useful information for initiating clinical actions to ameliorate risk [6], [8]. Such re-evaluations could be used to determine whether treatments provided or prescribed at the initial hospitalization resolved risks to the patient during the follow-up period, or whether further changes or additions to patient treatment plans are needed. This could lead to changes in medications, changes in medication doses, evaluation of new diagnostic tests, increasing the frequency of patient re-evaluations, or recommending new treatments. That information could also provide an inexpensive but poignant opportunity to counsel patients on compliance with lifestyle changes and medication adherence. This repeated measurement approach to risk evaluation and modification is standard practice today for the treatment of hyperglycemia, hyperlipidemia, hypertension, and tobacco addiction, and for IMRS could take a very similar approach.

The lack of data regarding treatments that will reduce IMRS and populations that are most in need of monitoring is an inhibitor to patient monitoring and managed care based on IMRS. Since glucose and creatinine are included in IMRS, therapies that reduce these biomarkers have the potential to reduce IMRS. Indeed, a decrease in IMRS from baseline to the second laboratory measurements was found among patients undergoing digestive and kidney surgeries at baseline. Furthermore, lower IMRS was also found for procedures or surgeries of the eye/ear/nose, lung surgeries and procedures, other respiratory treatments, ventricular assist device implantation, blood vessel surgeries and procedures, other hemic/lymphatic treatments, and urinary, reproductive, obstetrical, and miscellaneous surgeries and procedures, as well as anti-depressant medications. Possible treatments that may reduce CBC and BMP values but that did not achieve statistical significance included cardiac valve and coronary bypass surgeries, and beta-blockers, diuretics, and bronchodilator medications.

A handful of treatments, including PCI, other cardiac surgeries (those not specifically mentioned), ACE inhibitors, ARBs, anti-platelet agents, and warfarin were actually associated with an increase in IMRS between the first and second IMRS measurements. These associations likely reveal the continuation of adverse health due to smoldering, unresolved medical conditions rather than that these treatments increase risk. The data may, however, indicate the patient populations in which the opportunity for further risk-reduction is the greatest. All of these potential changes in IMRS should be further evaluated to validate the associations found herein and to determine whether altering patient management based on information from IMRS can resolve patient risk.

This study is potentially limited by the observational nature of the study, including because of the heterogeneity of setting for the second laboratory tests for IMRS calculation, thus the study may have unmeasured issues related to observational designs. Further prospective validation of the study’s findings is indicated.

### Conclusions

Computed IMRS values (http://intermountainhealthcare.org/IMRS/) both at follow-up (i.e., an average of one year after hospitalization) and at baseline were independently associated with mortality and cardiovascular events. Repeated measurement of IMRS provided enhanced ability to risk-stratify patients beyond measuring it at a single time point, both for mortality and for incident cardiovascular events. RDW at baseline and follow-up, as well as other CBC and BMP values, were also independently predictive of mortality and cardiovascular events. Further study of methods of clinical patient management based on IMRS is needed to evaluate IMRS as a potential tool for clinical risk adjustment [8]. This study also provided a glimpse of specific populations in which particular attention to risk level may be needed and for whom changes in risk may provide the most opportunity to reduce risk, but these hypotheses require further study in a randomized clinical trial framework.

## Supporting Information

Figure S1 **Association of baseline and follow-up IMRS metrics with incident MI, HF, and stroke in bivariable Cox regression analyses among A) females and B) males.** The referent group in analyses of baseline IMRS was low-risk baseline IMRS and in analyses of follow-up IMRS was low-risk follow-up IMRS.
(PDF)

Table S1 **ICD-9 codes for procedures and surgeries described in Table S4.**
(XLSX)

Table S2 **Change in IMRS (ΔIMRS) stratified by receipt of or absence of specified baseline treatments (for treatment definitions, see** **Table 1** **).**
(XLSX)

Table S3 **Multivariable Cox regression model of the association of all of the indicated follow-up and baseline CBC and BMP components with mortality among females.**
(XLSX)

Table S4 **For males, the multivariable Cox regression model of the association of the CBC and BMP components with mortality.**
(XLSX)

Appendix S1 **Reprint of** **Table 2** **from reference 1.**
(DOC)
