# Supplementary figures and images for: Repeated Measurement of the Intermountain Risk Score Enhances Prognostication for Mortality
Source: PLoS One. 2013 Jul 17;8(7):e69160. doi: 10.1371/journal.pone.0069160 (PMC3714235; doi:10.1371/journal.pone.0069160)

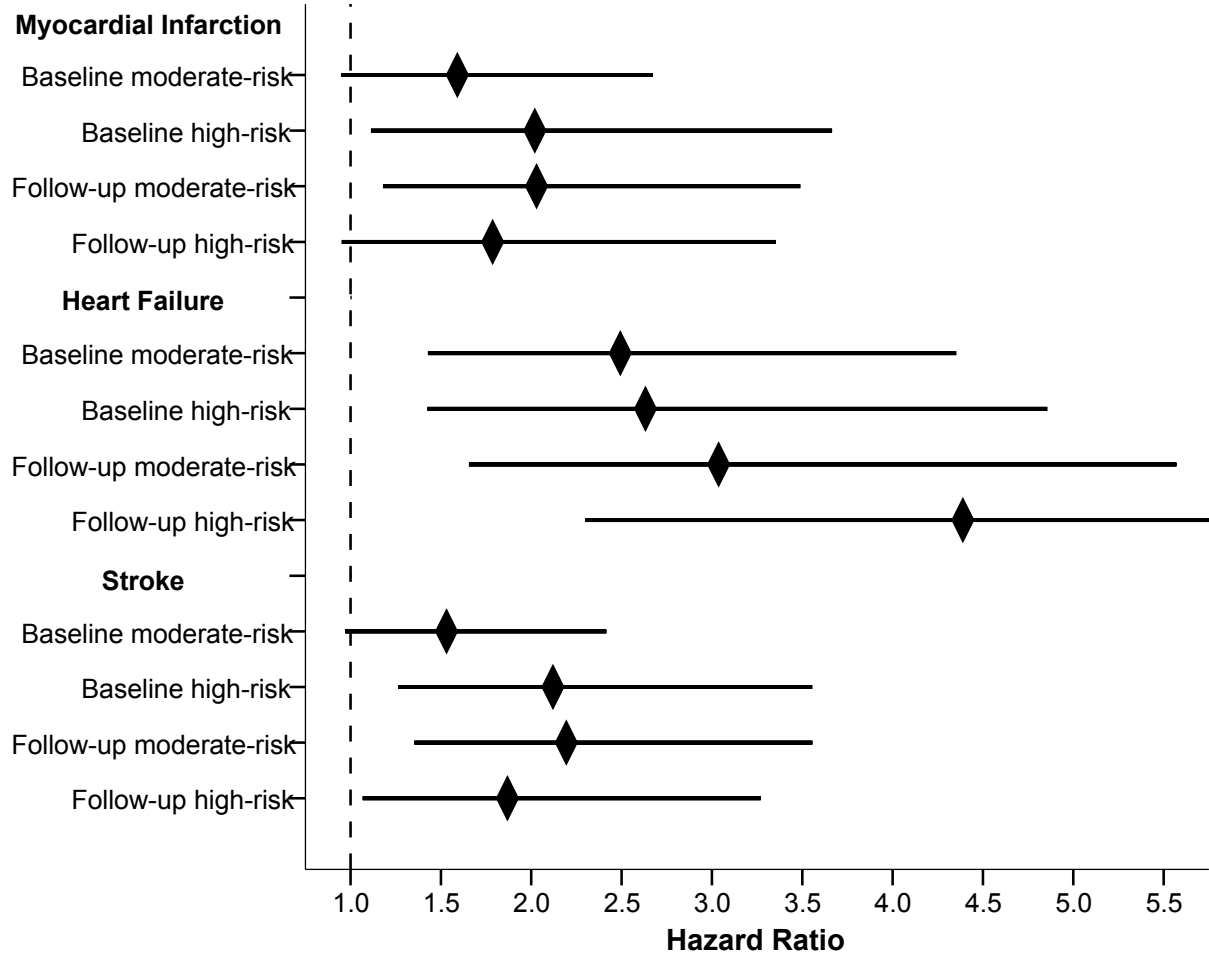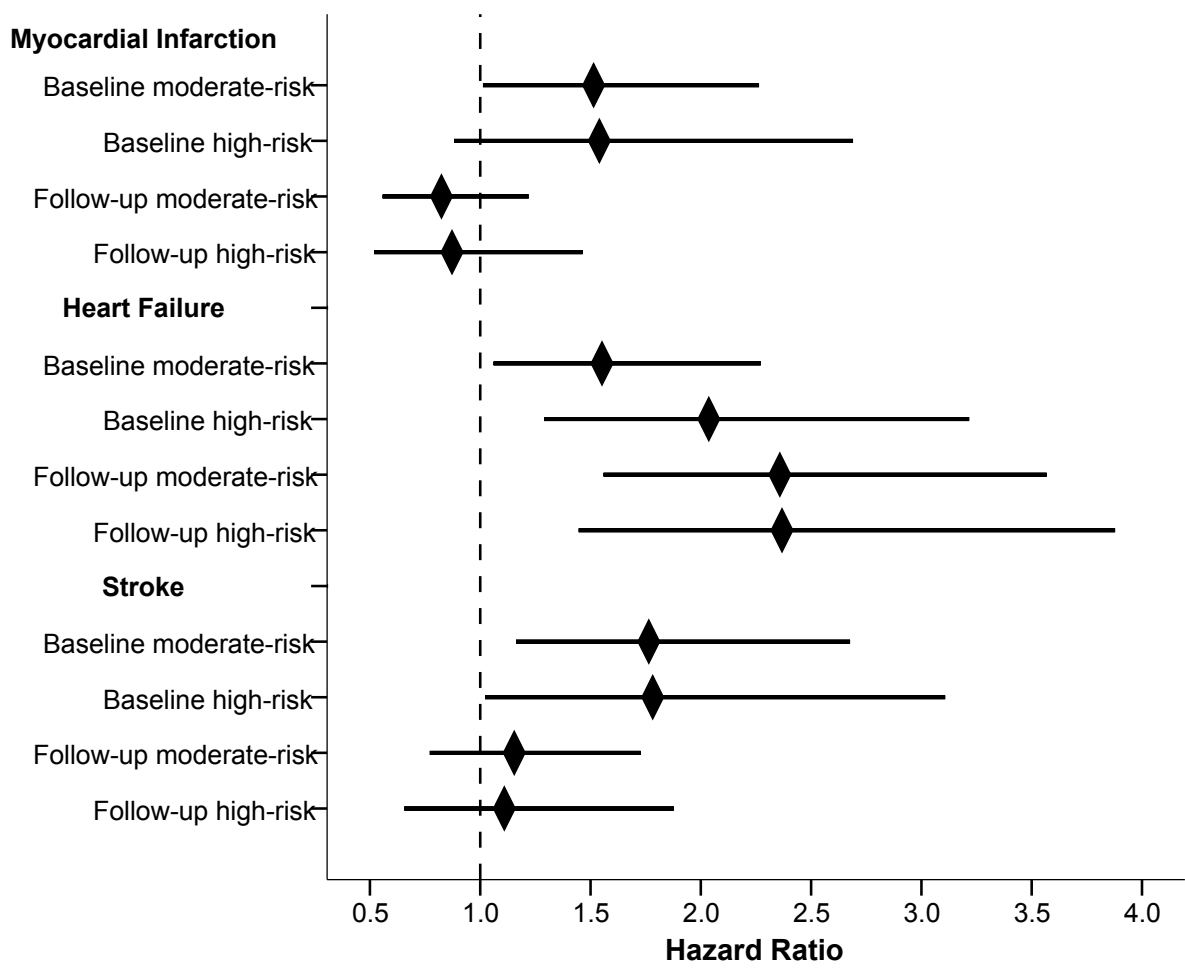

Supplement: Figure S1 — Association of baseline and follow-up IMRS metrics with incident MI, HF, and stroke in bivariable Cox regression analyses among A) females and B) males. The referent group in analyses of baseline IMRS was low-risk baseline IMRS and in analyses of follow-up IMRS was low-risk follow-up IMRS. (PDF) [file pone.0069160.s001.pdf]
