# Supplementary material for: Repeated Measurement of the Intermountain Risk Score Enhances Prognostication for Mortality
Source: PLoS One. 2013 Jul 17;8(7):e69160. doi: 10.1371/journal.pone.0069160 (PMC3714235; doi:10.1371/journal.pone.0069160)
Supplement: Appendix S1 — Reprint of Table 2 from reference 1. (DOC) [file pone.0069160.s006.doc]

APPENDIX S1:

Reprinted from the American Journal of Medicine, 2009/122, Benjamin D. Horne, Heidi T. May, Joseph B. Muhlestein, Brianna S. Ronnow, Donald L. Lappé, Dale G. Renlund, Abdallah G. Kfoury, John F. Carlquist, Patrick W. Fisher, Robert R. Pearson, Tami L. Bair, Jeffrey L. Anderson, Exceptional Mortality Prediction by Risk Scores from Common Laboratory Tests, Pages 550-558, Copyright (2009), with permission from Elsevier.

<http://www.sciencedirect.com/science/journal/00029343>

doi:10.1016/j.amjmed.2008.10.043

| **Table 2**. Sex-specific values* are used to calculate the Intermountain Risk Score as the sum of an individual’s corresponding values from each component at a given time point. | | | | | | | |
| --- | --- | --- | --- | --- | --- | --- | --- |
|  |  |  |  |  |  |  |  |
|  | Females | | |  | Males | | |
| **Component** | **30-day** | **1-year** | **5-year** |  | **30-day** | **1-year** | **5-year** |
| Hematocrit |  |  |  |  |  |  |  |
| ≤34.6 | 1 | 1 | 2 |  | 2 | 3 | 3 |
| 34.7-38.2 | 0 | 0 | 1 |  | 2 | 2 | 3 |
| 38.3-41.0 | 0 | 0 | 0 |  | 1 | 1 | 2 |
| 41.1-44.1 | 0 | 0 | 0 |  | 0 | 1 | 1 |
| ≥44.2 | 0 | 0 | 1 |  | 0 | 0 | 0 |
| White Blood Cell Count |  |  |  |  |  |  |  |
| ≤5.9 | 0 | 0 | 0 |  | 0 | 1 | 0 |
| 6.0-7.3 | 0 | 0 | 0 |  | 0 | 0 | 0 |
| 7.4-8.9 | 1 | 0 | 0 |  | 0 | 1 | 1 |
| 9.0-11.2 | 2 | 1 | 1 |  | 2 | 2 | 1 |
| ≥11.3 | 4 | 3 | 2 |  | 4 | 3 | 2 |
| Platelet Count |  |  |  |  |  |  |  |
| ≤183 | 2 | 1 | 2 |  | 2 | 1 | 1 |
| 184-220 | 1 | 0 | 0 |  | 1 | 0 | 0 |
| 221-254 | 1 | 0 | 1 |  | 0 | 0 | 0 |
| 255-300 | 0 | 0 | 1 |  | 1 | 1 | 0 |
| ≥301 | 0 | 0 | 1 |  | 1 | 1 | 1 |
| Mean Corpuscular Volume |  |  |  |  |  |  |  |
| ≤86.3 | 0 | 0 | 0 |  | 0 | 0 | 0 |
| 86.4-89.1 | 0 | 0 | 0 |  | 0 | 0 | 0 |
| 89.2-91.4 | 1 | 0 | 0 |  | 0 | 0 | 0 |
| 91.5-94.0 | 0 | 0 | 1 |  | 0 | 0 | 0 |
| ≥94.1 | 1 | 1 | 1 |  | 1 | 1 | 1 |
| Mean Corpuscular Hemoglobin Concentration | | |  |  |  |  |  |
| ≤33.3 | 1 | 1 | 0 |  | 1 | 1 | 0 |
| 33.4-33.8 | 0 | 0 | 0 |  | 0 | 1 | 0 |
| 33.9-34.2 | 1 | 0 | 0 |  | 0 | 0 | 0 |
| 34.3-34.6 | 0 | 0 | 0 |  | 0 | 0 | 1 |
| ≥34.7 | 0 | 0 | 0 |  | 0 | 0 | 1 |
| Red Cell Distribution Width |  |  |  |  |  |  |  |
| ≤12.5 | 0 | 0 | 0 |  | 0 | 0 | 0 |
| 12.6-13.0 | 2 | 1 | 1 |  | 1 | 0 | 0 |
| 13.1-13.5 | 1 | 1 | 2 |  | 1 | 1 | 2 |
| 13.6-14.3 | 3 | 2 | 2 |  | 2 | 2 | 3 |
| ≥14.4 | 4 | 4 | 5 |  | 3 | 3 | 4 |
| Mean Platelet Volume |  |  |  |  |  |  |  |
| ≤7.5 | 1 | 1 | 1 |  | 1 | 1 | 0 |
| 7.6-8.0 | 1 | 0 | 1 |  | 1 | 0 | 0 |
| 8.1-8.4 | 1 | 0 | 0 |  | 2 | 0 | 0 |
| 8.5-9.1 | 0 | 0 | 0 |  | 0 | 0 | 0 |
| ≥9.2 | 0 | 0 | 0 |  | 1 | 0 | 0 |
| Sodium |  |  |  |  |  |  |  |
| ≤138 | 1 | 1 | 2 |  | 1 | 1 | 2 |
| 139 | 0 | 0 | 1 |  | 1 | 0 | 0 |
| 140-141 | 0 | 0 | 1 |  | 0 | 0 | 0 |
| 142 | 0 | 0 | 0 |  | 1 | 0 | 0 |
| ≥143 | 1 | 1 | 0 |  | 2 | 1 | 0 |
| Potassium |  |  |  |  |  |  |  |
| ≤3.7 | 1 | 1 | 1 |  | 2 | 0 | 0 |
| 3.8-3.9 | 0 | 0 | 0 |  | 1 | 0 | 0 |
| 4.0-4.1 | 0 | 0 | 1 |  | 1 | 0 | 0 |
| 4.2-4.4 | 0 | 0 | 0 |  | 0 | 0 | 0 |
| ≥4.5 | 1 | 0 | 1 |  | 1 | 0 | 0 |
| Bicarbonate |  |  |  |  |  |  |  |
| ≤23 | 3 | 1 | 1 |  | 4 | 2 | 1 |
| 24-25 | 1 | 0 | 0 |  | 2 | 0 | 0 |
| 26 | 1 | 0 | 0 |  | 1 | 0 | 0 |
| 27-28 | 0 | 0 | 0 |  | 0 | 0 | 0 |
| ≥29 | 2 | 1 | 1 |  | 1 | 1 | 1 |
| Calcium |  |  |  |  |  |  |  |
| ≤8.5 | 4 | 3 | 3 |  | 1 | 2 | 2 |
| 8.6-8.9 | 2 | 2 | 2 |  | 0 | 1 | 2 |
| 9.0-9.2 | 2 | 1 | 1 |  | 0 | 0 | 1 |
| 9.3-9.5 | 0 | 0 | 0 |  | 0 | 1 | 0 |
| ≥9.6 | 1 | 1 | 0 |  | 0 | 0 | 0 |
| Glucose |  |  |  |  |  |  |  |
| ≤85 | 1 | 0 | 0 |  | 1 | 1 | 0 |
| 86-94 | 0 | 0 | 0 |  | 0 | 0 | 0 |
| 95-104 | 1 | 0 | 1 |  | 1 | 1 | 0 |
| 105-125 | 1 | 1 | 1 |  | 2 | 1 | 1 |
| ≥126 | 3 | 2 | 2 |  | 3 | 2 | 1 |
| Creatinine |  |  |  |  |  |  |  |
| ≤70.7 | 0 | 1 | 1 |  | 2 | 3 | 2 |
| 70.8-79.6 | 0 | 0 | 1 |  | 1 | 1 | 1 |
| 79.7-88.4 | 0 | 0 | 0 |  | 0 | 1 | 0 |
| 88.5-106.1 | 1 | 1 | 1 |  | 0 | 0 | 0 |
| ≥106.2 | 2 | 2 | 3 |  | 2 | 2 | 1 |
| Age (years) |  |  |  |  |  |  |  |
| 18-29 | -3 | -5 | -5 |  | 1 | 0 | 0 |
| 30-39 | -2 | -1 | -1 |  | 1 | -1 | 0 |
| 40-49 | 0 | 0 | 0 |  | 0 | 0 | 0 |
| 50-59 | 1 | 1 | 1 |  | 1 | 1 | 1 |
| 60-69 | 2 | 2 | 3 |  | 1 | 1 | 2 |
| 70-79 | 2 | 3 | 4 |  | 2 | 2 | 3 |
| ≥80 | 5 | 6 | 8 |  | 4 | 5 | 7 |
| Sex |  |  |  |  |  |  |  |
| Female | 0 | 0 | 0 |  | ----- | ----- | ----- |
| Male | ----- | ----- | ----- |  | 0 | 0 | 0 |
|  |  |  |  |  |  |  |  |
| *Risk models and component values are Copyright © 2006-2008, IHC Health Services, Inc. (freely available for academic use). | | | | | | | |
